# ROAD DIETS IMPACT ON DRIVING BEHAVIOR AMONG OLDER ADULTS WITH AND WITHOUT PRECLINICAL ALZHEIMER’S PATHOLOGY

**DOI:** 10.1093/geroni/igad104.3578

**Published:** 2023-12-21

**Authors:** David Carr, Julie Wisch, Beau Ances, Ganesh Babulal

**Affiliations:** Washington University in St. Louis, St. Louis, Missouri, United States; Washington University School of Medicine, St. Louis, Missouri, United States; Washington University School of Medicine, St. Louis, Missouri, United States; Washington University School of Medicine, St. Louis, Missouri, United States

## Abstract

Road diets (that is, the reallocation of one or more lanes of car traffic to other uses) have been proposed as a modification to increase pedestrian safety, particularly for older adults. We considered the impacts of road diets on aging drivers, and on those with early pathological accumulation of AD. We observed naturalistic driving data for 60 cognitively normal older drivers (Age Range = 62 – 87 years, Median = 75 years) driving across three road segments located in St. Louis, Missouri. We used neuroimaging and lumbar puncture derived biomarker data to determine which of the drivers had preclinical AD. Since previous AD studies identified a variety of changes in driving behavior among older drivers with preclinical AD, we examined driving speed before and after lane repurposing. We found that drivers with preclinical AD drove at lower speeds compared to those without preclinical AD prior to road diet implementation. After lanes were repurposed, there was no statistical difference in the speed between older drivers with and without preclinical AD. We evaluated cognitive performance and found that attentional control had a mediating effect on driver speed, suggesting that an individual’s ability to focus on a specific task and filter out distractions was associated with faster driving. Driving speed after lane repurposing is not mediated by attentional control, suggesting that road diets are impervious to individual driver capacity. We conclude that lane repurposing has potential as an important mobility infrastructure solution that could enhance older driver safety and facilitate aging in place.

